# Trait emotional intelligence and self-assessment of classroom learning in medical students

**DOI:** 10.30476/jamp.2020.84674.1147

**Published:** 2020-07

**Authors:** Himel Mondal, Shaikat Mondal

**Affiliations:** 1 Department of Physiology, Bhima Bhoi Medical College and Hospital, Balangir, Odisha, India; 2 Department of Physiology, Raiganj Government Medical College and Hospital, West Bengal, India

**Keywords:** Academic performance, Emotions, Emotional intelligence, Medical student, Self-assessment, Self-control

## Abstract

**Introduction:**

Trait emotional intelligence (EI) is the self-perception of emotional abilities. It is an important predictor of academic performance. Students’ self-assessment (SSA) of knowledge gained from classroom teaching may help in the identification of deficiencies in knowledge and provide scope for further improvement. We aimed to evaluate the correlation between EI and SSA capability.

**Methods:**

We conducted a cross-sectional, observational study with 56 first-year medical students recruited as a convenience sample. We used the “Trait Emotional Intelligence Questionnaire Short Form” to assess Trait EI. For assessment of SSA, we asked the participants to write answers to a set of questions related to the topic of the preceding 1-h lecture and to assess their marks themselves. Three subject experts checked the answer sheets and we took the mean as the expert assessment (EA) marks. The correctness score of prediction was calculated by comparing SSA and EA marks. Pearson correlation coefficient (r) was calculated between EI scores and SSA correctness score.

**Results:**

In all sessions, the students underpredicted their marks. SSA correctness score showed a positive correlation with well-being (r=0.33; P=0.01); self-control (r=0.57; P<0.01); emotionality (r=0.51; P<0.01); sociability (r=0.51; P<0.01); and total score (r=0.64; P<0.01) of trait EI.

**Conclusion:**

Underprediction of marks in formative assessment is common in 1st-year medical students. Students with higher levels of EI may predict their knowledge gained from classroom better than the students with lower EI. This may be a potential reason for the better academic performance of students with higher EI.

## Introduction

Emotional intelligence (EI) is “the capacity to be aware of, control, and express one's emotions, and to handle interpersonal relationships judiciously and empathetically”( 1 , 2 ). There are two different constructs of EI; one is interpersonal, and the other is intrapersonal. The interpersonal construct of EI, designated as “ability EI” reflects the person’s ability to understand other people. In contrast, the intrapersonal construct, designated as “trait EI” is concerned with self-perception of emotional abilities and it can be assessed by a self-report questionnaire ( 3 ).

EI has a positive correlation with academic performance, especially, the capability of assessing self-emotion, which is a component of trait EI ( 4 , 5 ). Aithal *et al*. reported that credit-group students (students securing 65-74% marks) and distinction-group students (students securing 75-84% marks) have higher EI than pass-group students (students securing 50-64% marks) ( 6 ). In addition, Chew *et al*. found that in both continuous and final assessments, students with higher EI perform better than their peer with lower EI ( 7 ).

Assessment of academic performance can be mainly of three types - “expert assessment” by qualified experts (i.e., teachers), “peer assessment” by classmates, and “self-assessment” by the students themselves. These three types of assessment may show a difference in score as the evaluation is subjective ( 8 , 9 ). Students’ self-assessment (SSA) of academic performance is helpful for academic self-regulation ( 10 ). It helps in identification of the gap in academic performance ( 11 ). The major advantage of self-assessment is that it can be done by the students themselves without the help of others. If the students can assess their knowledge on a particular topic properly, they can reallocate or regulate effort and time for a balanced improvement in all subjects. 

Self-assessment should preferably be checked with the feedback from the experts for a better understanding as there may be inaccurate self-assessment ( 12 , 13 ). Otherwise, the aim of SSA would fail. Over-prediction of marks (e.g., student predicted mark is 50, but the expert-assessed mark is 20) would divert the focus from the topic and under-prediction may divert all the concentration to that particular topic, ignoring others. Hence, an optimum level of agreement between SSA and expert assessment is needed for academic improvement ( 14 ).

Currently, available literature has established that the higher the EI, the higher the academic performance ( 4 , 7 ). In addition, implementation of SSA has been shown to increase academic performance ( 14 ). However, there is a gap in the literature about the relationship between EI and SSA. Hence, we conducted this pilot study to explore any correlation between trait EI and correctness of SSA of academic performance.

## Methods

This cross-sectional observational study was conducted in Fakir Mohan Medical College, Balasore, Odisha, India. The college is a government-run medical college with an annual intake of 100 medical students. We conducted this study between August 2018 and August 2019.

This was a questionnaire-based study. The participants of this study were exposed to negligible risks. After briefing about the aim and protocol of the study, the participants providing written consent were included in the study. After taking permission from the institution, this study was conducted in full accordance with the Declaration of the Helsinki (64^th^ World Medical Association General Assembly, Fortaleza, Brazil, October 2013).

To the best of our knowledge, no previous study was conducted with the same aim and assessment tools. Hence, for calculating the minimum sample size, we expected a correlation coefficient between the total score of trait EI and SSA correctness score to be ±0.5. With this assumption and α=0.05, and the power of the study 95%, the minimum calculated sample size was 46 ( 15 , 16 ). Adding 20% for expected high dropout and high chances of missing data, the final sample size was 55. The study was carried out with a “convenience sample” where an inclusion criterion was any first-year medical student of the college providing written consent for participation. There were no exclusion criteria.

**Figure 1:**
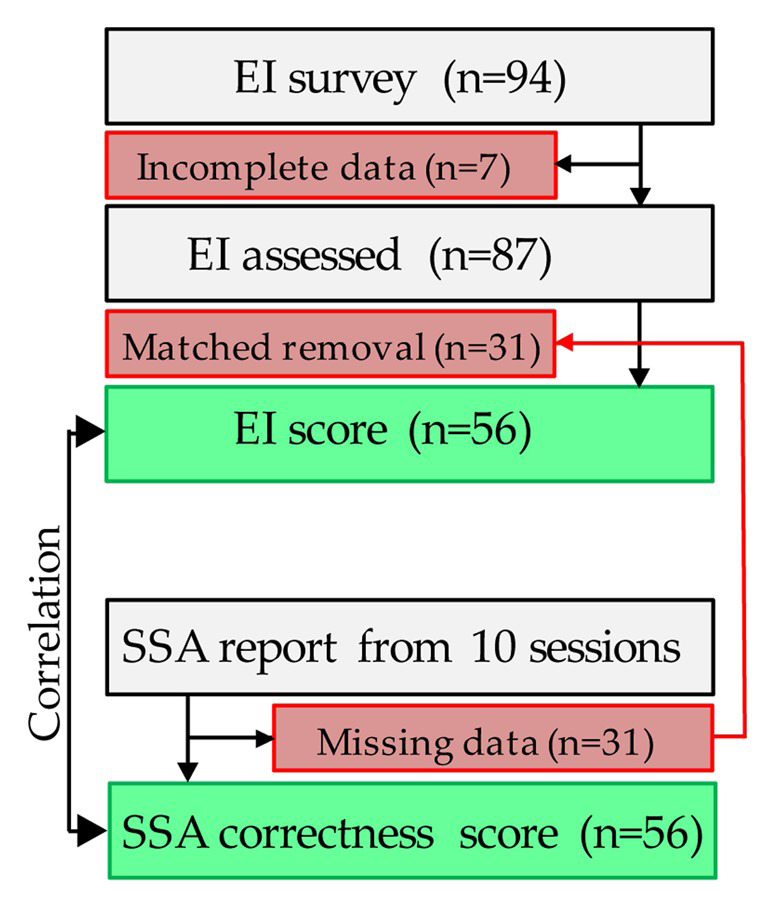
Sample size and brief study flow chart

First, we conducted a questionnaire-based survey for assessing the EI. Among the responses, seven forms were incomplete. Hence, EI of total 87 students was preserved for further analysis. Then we conducted 10 sessions of self-assessment of knowledge gained from 1-h lectures. Some students were absent in one or more session of the SSA. Only those who completed 10 sessions were included in the final analysis. After getting the SSA data, we removed the subjects who were absent in any session of 10 formative assessments. The sample in each stage of the survey and assessment is shown in Figure 1.

Footnote: In each session of 10 formative assessments, a different set of students participated. Hence, the sample size (n) is not mentioned

For measuring EI, we used the “Trait Emotional Intelligence Questionnaire – Short Form (TEIQue-SF)” ( 17 ). This instrument measures emotional intelligence in “well-being”, “self-control”, “emotionality”, and “sociability” dimensions with a global score of trait EI. The questionnaire consists of 30 statements in English and each statement has 7-point Likert-type response option (completely disagree = 1 to completely agree = 7 without defining other points in between). All participants in this study were capable of speaking and writing English. We conducted a pre-testing and cognitive interview about the understanding of the questionnaire on five students to ascertain its feasibility for administration. With the experience and students’ input, we added two easily understandable synonyms for the word “gloomy (statement 12)” and “negotiator (statement 21)” on the questionnaire after consulting two language experts.

First, we briefed the students about the questionnaire and response options. Then, we distributed the questionnaire among them and instructed them to fill the responses themselves as quickly as possible.

When we commenced the study (2018) and collected the questionnaire, the questionnaire and the scoring key were available free for non-commercial academic usage on the Psychometric Laboratory ( 18 ). TEIQue-SF needs direct scoring for the responses for positive statements (e.g., “I can deal effectively with people”). The responses of negative statements (e.g., “I generally don’t find life enjoyable”) needs reverse scoring (i.e., score 7 = score 1, score 6 = score 2, score 5 = score 3, score 3 = score 5, score 2 = score 6, score 1 = score 7). We coded the response accordingly on a spread-sheet manually. Now, the scoring key is not available on the website, and the scoring can be done only on the website homepage. Hence, despite doing the calculation manually, we cannot share the details of the calculation here. Any researcher, for any future study, may get the scoring done from the website of London Psychometric Laboratory ( 18 ).

Students’ engagement during collection attendance (SEdCA) is a method of collecting attendance where the teacher asks the students to write answers to a set of questions related to the lecture topic at the end of the 1-h lecture. The students write the answers on a sheet with their roll number and name. The attendance is recorded from the answer sheets. This method was introduced for collection of attendance. Its application in formative assessment has also been established ( 19 ). In this study, we used the SEdCA as a method of formative assessment. The students were instructed to write the answer and provide the self-assessed marks on the sheet. The students had 10 marks for each session and we conducted 10 sessions of formative assessments. The self-assessed marks were considered as the SSA.

Three expert evaluators checked the answer sheets of the same 10 sessions for formative assessment. We took the average marks of the three evaluators as the final “Expert assessment (EA)”. The Percent Error Score (PES) was calculated to find the direction of error from the following formula:

$\mathrm{PES}=\frac{(\mathrm{SSA}-\mathrm{EA})\times 100}{\mathrm{EA}}$

 Where, PES = Percent error score, SSA = Students’ self-assessment, and EA = Expert assessment

In this formula, a positive sign indicates over-prediction and a negative sign indicate under-prediction.

Correctness of assessment of a session was calculated by the following formula:

$\mathrm{CS}=1-\frac{\left|\mathrm{SSA}-\mathrm{EA}\right|}{\mathrm{EA}}$

Where, CS = Correctness score, SSA = Students’ self-assessment, and EA = Expert assessment

Each student had CS for 10 sessions and the average of the 10 sessions was considered the "SSA average correctness score." Henceforth, the SSA correctness score of a student indicates the average correctness score.

The data were expressed in mean and standard deviation. Variables in males and females were compared with unpaired t-test with two-tail α = 0.05. For categorizing subjects with a correct prediction, a deviation of prediction within ±9.99% of EA was calculated (i.e., if EA mark is X and SSA mark is Y, then Y would be considered correct if it is within “X ± 9.99% of X”). From the direction obtained by PES, the students were categorized as “correct prediction”, “over-prediction”, and “under-prediction” groups. The categorical data were statistically tested by the Chi-Square test. The Pearson correlation coefficient (r) was calculated with the correctness score and 4 dimensions of trait EI and global EI with two tail α=0.05. The statistical analysis was carried out in Microsoft Excel® and GraphPad Prism 6.01 (GraphPad Software, Inc., CA, USA).

## Results

The data from 56 participants with the mean age 17.96±0.87 (range: 17-20) years were analysed. Overall and sex-wise measured variables are presented in Table 1. There was no difference of EI and SSA correctness scores between males and females.

**Table 1:** Age, trait emotional intelligence score, and students’ self-assessment correctness score

Variables		Overall (n=56)	Female (n=35)	Male (n=21)	t	P
		Mean±SD, Range				
Age (years)		17.96±0.87, 17-20	17.94±0.84, 17-20	18±0.95, 17-20	0.24	0.82
Trait EI score (1-7)	Wellbeing	5.38±0.9, 2.83-6.83	5.32±0.94, 3.33-6.83	5.47±0.84, 2.83-6.67	0.58	0.56
	Self-control	4.47±1.02, 1.66-6	4.6±0.94, 2.16-6	4.27±1.13, 1.66-5.5	1.16	0.25
	Emotionality	4.77±0.86, 3.13-6.5	4.65±0.83, 3.13-6	4.98±0.89, 3.38-6.5	1.37	0.18
	Sociability	4.43±0.93, 1.33-6.83	4.45±0.93, 2.33-6.83	4.4±0.97, 1.33-6.33	0.2	0.85
	Global	4.8±0.69, 2.8-6.27	4.77±0.68, 2.96-6.27	4.85±0.73, 2.8-5.67	0.42	0.68
SSA average correctness score		0.84±0.09, 0.54-1	0.85±0.09, 0.59-1	0.83±0.1, 0.54-1	0.68	0.5

SD: Standard deviation, EI: Emotional intelligence, SSA: Students’ self-assessment

The number of students predicting marks of formative assessment is shown in Table 2. In all sessions, the majority of the students under-predicted the marks. However, the difference in formative assessment number 5, 8, and 10 was not statistically significant. In addition, we calculated the correlation between SSA marks and EA marks. The correlation was r = 0.68 (95% CI: 0.51 - 0.80, R2 = 0.46, P <0.01).

**Table 2:** Correct, over, and under-prediction of marks in 10 sessions of formative assessment

Session	Correct prediction	Over prediction	Under prediction	χ2	P
	n (%)				
1	17 (30.36)	8 (14.29)	31 (55.36)	14.39	<0.01*
2	14 (25)	11 (19.64)	31 (55.36)	12.46	<0.01*
3	19 (33.93)	7 (12.5)	30 (53.57)	14.18	<0.01*
4	15 (26.79)	8 (14.29)	33 (58.93)	17.82	<0.01*
5	21 (37.5)	13 (23.21)	22 (39.29)	2.61	0.27
6	17 (30.36)	9 (16.07)	30 (53.57)	12.04	<0.01*
7	15 (26.79)	12 (21.43)	29 (51.79)	8.82	0.01*
8	22 (39.29)	13 (23.21)	21 (37.5)	2.61	0.27
9	16 (28.57)	11 (19.64)	29 (51.79)	9.25	<0.01*
10	20 (35.71)	14 (25)	22 (39.29)	1.86	0.39

* Statistically significant P-value of Chi-square test

SSA correctness score and its correlation with trait EI are shown in Table 3. All dimensions showed a statistically significant positive correlation with the correctness score. Global (i.e., total) score of EI showed the highest positive correlation (r = 0.64, P <0.01) when compared to the four dimensions of trait EI.

**Table 3:** Correlation of students’ self-assessment correctness score and trait emotional intelligence score

Statistics	Trait EI				
	Wellbeing	Self-control	Emotionality	Sociability	Total score
r	0.33	0.57	0.51	0.51	0.64
95% CI	0.07 - 0.55	0.36 - 0.72	0.28 - 0.68	0.29 - 0.68	0.45 - 0.77
R^2^	0.11	0.32	0.26	0.26	0.41
P	0.01	<0.01	<0.01	<0.01	<0.01

r: Pearson correlation coefficient, CI: Confidence interval, R2: Coefficient of determination

## Discussion

We found a positive correlation between SSA and EI. All trait EI dimensions showed a positive correlation with the SSA correctness score. Among the dimensions, “self-control” showed the highest positive correlation and “wellbeing” showed the lowest positive correlation. Hence, medical students with a higher level of overall trait EI, and especially of stronger “self-control,” may predict their marks in formative assessment better than those with relatively lower EI. As the students with higher EI can assess their knowledge on a particular topic, this would enable them to improve their performance accordingly. This finding is a new addition to the current literature.

The higher EI is associated with better academic performance in Indian medical, dental, and nursing students ( 20 - 22 ). The EI of a student is not a constant attribute and it can be improved by long-term training ( 23 ). Hence, a properly designed training program may help in the improvement of EI. As we found a positive correlation between SSA and EI, an improvement in EI may also increase the SSA capability. However, whether improvement of EI improves SSA accuracy in a cohort was beyond the scope of this study. This may be a topic of future research. 

The majority of the students in this study under-predicted their marks in formative assessment. This finding is corroborative with the finding by Papinczak *et al*. ( 12 ). In contrast, Saban *et al*. showed that the majority of United Arab Emirates medical students over-predicted their examination marks ( 24 ). Over-prediction is also seen in Iranian nursing students ( 25 ). This discordant finding among different studies may be attributed to different sample of students and usage of different assessment criteria ( 26 ). Prediction of marks is an important aspect in self-assessment as a proper prediction would guide the students for balanced improvement.

We found an equal level of EI in male and female first-year medical students. Meshkat *et al*. also showed that there is no gender difference in EI in the Iranian undergraduate language learners ( 27 ). In addition, Pardeller *et al*. and Vasefi *et al*. also showed similar results ( 28 , 29 ). In contrast, Aithal *et al*. showed that Indian female medical students had more EI than male counterparts ( 6 ). The potential reason for this disagreement may be due to the sample of different geographical area and different assessment methods.

The strength of this study is the exploration of a novel relation between EI and SSA with adequate sample for a correlation study. The questionnaire for acquiring EI score was pre-tested on a sample and modified for better understanding. To reduce bias in expert assessment, we calculated average marks awarded by three experts. Formative assessments were carried out immediately after the 1-h lecture (what is taught) so that the students could assess their learning (what I learned from the lecture) without recall bias.

The major weakness of this study is that we explored only written formative assessment as the knowledge gained from classroom teaching. Choosing the sample conveniently from 1st-year medical students from a medical college was a limitation for generalization of the results to the entire Indian 1st-year medical students. However, we were bound by the time, labour, and fund for further extending the research to other institutions. This pilot study opens a new area of medical education to be explored in future studies.

## Conclusion

The majority of the first-year medical students underpredict their marks in formative assessments. Among the students, a higher EI can be considered as an indicator of higher accuracy of SSA of classroom learning. This may be a reason why students with higher EI perform better. They can judge their performance more effectively and adjust their improvement strategy. At an early stage of an academic session, EI can be assessed to help students with lower EI in their judgement and further improvement.
